# Ovarian sensitivity index is strongly related to circulating AMH and may be used to predict ovarian response to exogenous gonadotropins in IVF

**DOI:** 10.1186/1477-7827-9-112

**Published:** 2011-08-09

**Authors:** Valentina Biasoni, Ambra Patriarca, Paola Dalmasso, Angela Bertagna, Chiara Manieri, Chiara Benedetto, Alberto Revelli

**Affiliations:** 1 Physiopathology of Reproduction and IVF Unit, University of Torino, S.Anna Hospital, Torino, Italy; 2 Medical Statistics Unit, Department of Public Health and Microbiology, University of Torino, Torino, Italy; 3 Endocrinology Laboratory, Department. of Internal Medicine, A.O.U.S. Giovanni Battista, University of Torino, Italy

## Abstract

**Background:**

Serum anti-Mullerian hormone (AMH) is currently considered the best marker of ovarian reserve and of ovarian responsiveness to gonadotropins in in-vitro fertilization (IVF). AMH assay, however, is not available in all IVF Units and is quite expensive, a reason that limits its use in developing countries. The aim of this study is to assess whether the "ovarian sensitivity index" precisely reflects AMH so that this index may be used as a surrogate for AMH in prediction of ovarian response during an IVF cycle.

**Methods:**

AMH serum levels were measured in 61 patients undergoing IVF with a "long" stimulation protocol including the GnRH agonist buserelin and recombinant follicle-stimulating hormone (rFSH). Patients were divided into four subgroups according to the percentile of serum AMH and their ovarian stimulation was prospectively followed. Ovarian sensitivity index (OSI) was calculated dividing the total administered FSH dose by the number of retrieved oocytes.

**Results:**

AMH and OSI show a highly significant negative correlation (r = -0.67; *p *= 0.0001) that is stronger than the one between AMH and the total number of retrieved oocytes and than the one between AMH and the total FSH dose.

**Conclusions:**

OSI reflects quite satisfactory the AMH level and may be proposed as a surrogate of AMH assay in predicting ovarian responsiveness to FSH in IVF. Being very easy to calculate and costless, its use could be proposed where AMH measurement is not available or in developing countries where limiting costs is of primary importance.

## Background

In human in-vitro fertilization (IVF), the identification of variables able to predict ovarian responsiveness to exogenous gonadotropins allows to individualize treatment protocols optimizing results and reducing complications.

Over the last two decades, a number of clinical, endocrine and ultrasound-detectable parameters have been proposed as markers of ovarian follicular reserve and as predictors of ovarian responsiveness to hormonal stimulation. Most of them, unfortunately, have limited predictive value [1,2], that can be partially compensated by combining different variables in multi-marker prediction models [3].

Circulating anti-Mullerian hormone (AMH) is presently claimed to be the best marker of ovarian reserve and ovarian responsiveness to stimulation. In fact, it has resulted to be superior to patient's age, basal follicle-stimulating hormone (FSH), estradiol, inhibin B [4] and at least comparable to antral follicle count (AFC) [5] in predicting ovarian responsiveness to exogenous gonadotropins. AMH's predictive capacity on oocyte yield is well estabilished; differently, its significance in regard to oocyte quality and pregnancy potential is still under debate, even if recent work shows a good correlation between AMH levels and occurrence of a viable pregnancy [6].

AMH assay, however, is not yet available in all IVF labs and is quite expensive, a point that limits its use in developing countries, where particular attention must be paid to the economical aspects of IVF programs.

Ovarian sensitivity index (OSI) is an easily measurable parameter for patients previously submitted to at least one IVF. That is, it can be obtained dividing the total dose of exogenous gonadotropins by the number of oocytes retrieved at ovum pick-up (OPU).

Aim of the present study is to assess whether OSI accurately reflects AMH levels in IVF patients and therefore could be proposed as a surrogate of AMH measurement where AMH assay is not available.

## Methods

### Patients

The study included 61 women attending for the first time the IVF program at the Physiopathology of Reproduction and IVF Unit of S. Anna University Hospital between June 2009 and April 2010. All patients satisfied the following criteria: age ≤ 40 years; BMI between 19 and 30 kg/m^2^; basal (day 3) FSH ≤ 10 UI/l and AFC ≥ 8; regular menstrual cycles; no history of ovarian surgery, chemotherapy or pelvic irradiation; no evidence of endocrine disorders (normal prolactin, androgen and thyroid-stimulating hormone levels); no hormonal therapy in the six months preceding IVF. The study was authorized by the local ethical committee and a written informed consent was obtained from all recruited subjects.

### Ovarian stimulation protocol

In order to provide homogeneity to the study, all patients received a "long" gonadotropin-releasing hormone (GnRH)-agonist protocol. Buserelin (Suprefact, Hoechst, Germany) nasal spray (800 mcg/day) was administered for 10-14 days until complete pituitary desensitization was documented by US examination and estradiol measurement. Ovarian stimulation was then accomplished administering recombinant follicle-stimulating hormone (rFSH; Gonal F, Merck-Serono, Switzerland) at a daily dose between 100 and 300 UI that was individually established according to age, body mass index (BMI), basal FSH, and AFC (Table 1). Ovarian response to stimulation was monitored by transvaginal US examination plus serum estradiol measurement every second day from stimulation day 6. From day 6, the rFSH dose was adjusted according to ovarian response. When at least two leading follicles reached 18 mm diameter, a s.c. injection of 10.000 UI human chorionic gonadotropin (hCG; Gonasi HP, IBSA, Switzerland) was administered, and OPU was scheduled 36 hours later.

**Table 1:** Criteria to establish the starting daily dose of rFSH according to age, body mass index (BMI), basal FSH, and AFC

Clinical characteristics	rFSH starting dose (IU)
Age < 35 + normal BMI + basal FSH < 8 IU/L + AFC > 14	100 - 150
Age < 35 + BMI 25-30 + basal FSH < 8 IU/L + AFC > 14	200
Age 35-40 and/or basal FSH 8-10 IU/L and/or AFC 8-13	150 - 250
Age 35-40 and/or basal FSH 8-10 IU/L and/or AFC 8-13, BMI > 25	300

Patients above 40 years, with BMI > 30 or < 19, basal FSH levels > 10 IU/L and/or AFC < 8 were a priori excluded from the study.

OPU was performed by experienced operators that strictly adhered to the following criteria: (1) puncture all follicles > 10 mm diameter; (2) completely aspirate follicular fluid using a standard aspiration pressure (Rocket Aspiration Pump, USA); (3) signal every problem encountered during OPU that could have affected the chance to retrieve oocytes.

A single lumen Cook needle (Cook, Sydney, Australia) was used in all OPUs and follicular fluids were immediately given to the biologist for oocyte identification and retrieval. Using this procedure, oocytes were retrieved from approximately 95% of the punctured follicles.

### AMH measurement

A venous blood sample for AMH measurement was taken approximately one month before the scheduled IVF treatment, independently of the menstrual cycle phase. Blood samples were centrifuged at 3500 cycles/min for 10 min and sera were stored in sterile tubes and kept at -30°C for a time period not exceeding 2 months.

AMH was measured using a commercially available enzyme immunoassay kit (Immunotech Beckman Coulter, UK) following the manifacturer's instructions. The lowest detection limit of this assay is 0.14 ng/mL., whereas the maximal intra- and inter-assay coefficients of variation are 12.3% and 14.2%, respectively. In order to minimize the chance of bias in the assay, all sera were processed in double, during the same day, using the same measurement kits, and by the same operator.

### Ovarian sensitivity index (OSI) calculation

OSI was calculated by dividing the total administered rFSH dose by the number of oocytes retrieved at OPU, thus obtaining the FSH-to-retrieved oocyte ratio.

### Statistical analysis

Data were expressed as mean ± SD or counts and percentages. Patients were divided into four subgroups according to the circulating AMH level: below the 25^th ^percentile (AMH 0.25-1.1 ng/ml, subgroup A), between the 25^th ^and 50^th ^percentiles (AMH 1.2-1.6 ng/ml, subgroup B), between the 50^th ^and 75^th ^percentiles (AMH 1.7-2.6 ng/ml, subgroup C), and above the 75^th ^percentile (AMH 2.7-8.5 ng/ml, subgroup D).

Qualitative data were analyzed by means of Chi-square or Fisher's exact test. The normality assumption of the quantitative measures was verified by Shapiro-Wilk test and significance of between-group differences were assessed using ANOVA or Kruskal Wallis rank test, as appropriate. Pairwise comparisons of the groups were performed with Bonferroni's adjustment for multiple comparisons. Spearman's correlation test was used to test the relationship between OSI and patients' age, BMI, AFC and circulating AMH. To analyze the correlation between OSI and AMH a linear regression analysis adjusted by age was also performed.

All the tests were two-tailed tests with significance level set at 5%.

## Results

The mean age of the 61 patients included in the study was 34.9 ± 3.5 years and the mean BMI was 22.5 ± 2.9 kg/m^2^. Infertility causes were male factor (52.5% of cases), unexplained (26.2%), tubal damage (14.8%) and mixed factors (e.g. tubal factor plus male factor). The mean basal FSH level was 6.8 ± 1.7 IU/l, and the mean AFC was 15.2 ± 6.6. The mean AMH level in the patients' group was 2.3 ± 1.8 ng/ml.

The mean starting dose of rFSH was 198 ± 76 IU/day and the mean total rFSH dose was 2355 ± 1076 IU. The mean length of stimulation was 11.7 ± 1.7 days and the peak serum estradiol level the day of hCG administration was 2766 ± 1089 pg/ml. A mean number of 9.6 ± 4.5 oocytes were collected, 73% of which were meiotically mature. The overall fertilization rate was 91.8%, and 90.2% of the cycles leaded to embryo development and transfer in the uterus. Overall, the pregnancy rate per cycle was 32.7% and the implantation rate was 20%.

The four subgroups, formed on the basis of the AMH level, did not significantly differ for age, BMI, and infertility duration (Table 2). AFC was progressively higher from subgroup A to subgroup D; moreover, subgroup D had a mean AFC value significantly higher that the other subgroups (*p *< 0.0001). The number of retrieved oocytes in subgroup D was significantly higher than in subgroup A, but not significantly different vs. subgroups B and C. The total administered rFSH dose and the OSI showed the same trend of AFC, becoming progressively lower from subgroup A to subgroup D, and being significantly lower in subgroup D than in the other three subgroups (p < 0.0001) (Table 2).

**Table 2:** Clinical characteristics of patients subgrouped according to circulating AMH levels

	Subgroup A n = 14	Subgroup B n = 13	Subgroup C n = 18	Subgroup D n = 16	*p*	Significant comparisons
*MH (ng/ml)*	0.25-1.1	1.2-1.6	1.7-2.6	2.7-8.5		
*Age (years)*	35.8 ± 2.9	34.7 ± 3.7	34.8 ± 3.7	34.1 ± 3.8	0.64	
*BMI (Kg/m^2^)*	22.7 ± 3.4	21.7 ± 1.9	22.8 ± 3.0	22.8 ± 3.2	0.69	
*AFC*	10.3 ± 3.3	12.3 ± 3.6	14.6 ± 4.0	22.8 ± 6.9	0.0001*	D vs. A, B, C
*Total dose of rFSH (IU)*	3255 ± 1058	2418 ± 866	2347 ± 1066	1525 ± 527	0.0001*	D vs. A, B, C
*Stimulation length (days)*	12.5 ± 1.7	11.0 ± 1.2	11.7 ± 1.8	11.7 ± 2.0	0.17	
*N. of retrieved oocytes*	7.0 ± 3.0	8.6 ± 3.3	10.5 ± 4.9	11.8 ± 4.7	0.02*	A vs .D
*Ovarian sensitivity index (IU)*	798 ± 1128	337 ± 216	304 ± 319	153 ± 92	0.0001*	D vs. A, B, C

Subgroup A: below the 25^th ^percentile (AMH 0.25-1.1 ng/ml); Subgroup B: between 25^th ^and 50^th ^percentiles (AMH 1.2-1.6 ng/ml); Subgroup C: between 50^th ^and 75^th ^percentiles (AMH 1.7-2.6 ng/ml); Subgroup D: above the 75^th ^percentile (AMH 2.7-8.5 ng/ml).

**Figure 1:**
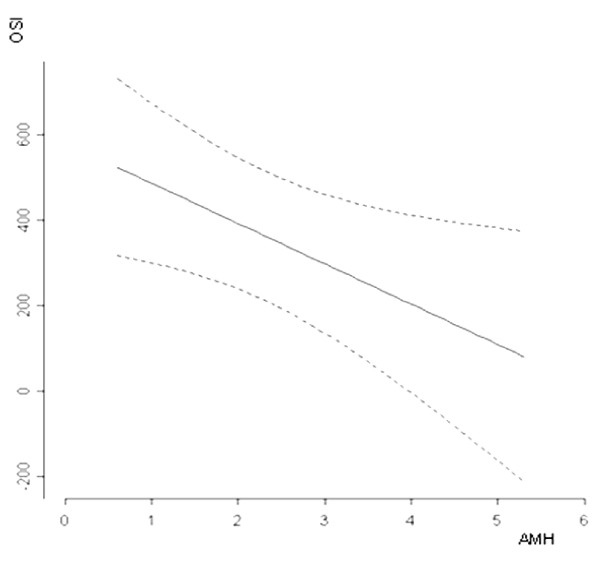
**Correlation (multiple regression analysis) between ovarian sensitivity index (OSI = FSH units per retrieved oocyte) and circulating AMH levels**. p = 0.028.

OSI was significantly correlated to age, but not to BMI; it was also inversely correlated to AFC levels (r = -0.59, *p *= 0.0001; Table 3). A highly significant inverse correlation was observed between OSI and AMH (r = -0.67, *p *= 0.0001), and was confirmed by the multivariate linear regression (p = 0.028; Figure 1). The correlation between OSI and circulating AMH that was stronger than those existing between AMH and the total number of oocytes (r = 0.41, p < 0.01), and between AMH and the total FSH dose (r = -0.49, p < 0.01).

**Table 3:** Correlation between ovarian sensitivity index (OSI = FSH units per retrieved oocyte) and patients' age, BMI, antral follicle count (AFC) and circulating AMH levels

	*r*	*p*
*Age*	0.40	0.0014
*BMI*	0.15	0.24
*AFC*	-0.59	0.0001
*AMH*	-0.67	0.0001

## Discussion

Ovarian stimulation is a key event in human IVF, and choosing properly the initial dose of gonadotropins may avoid a poor oocyte yield and, on the other side, an excessive response with high risk of ovarian hyperstimulation syndrome (OHSS). In most IVF Units, the individual dose to stimulate the ovary is tailored considering age, basal FSH level, ovarian volume and the count of small antral follicles (AFC). Prediction models that use these variables singularly or in combination reach an acceptable, even if not optimal, level of accuracy in estimating ovarian responsiveness to exogenous gonadotropins [1,3].

In the last years, serum AMH measurement has been introduced into the clinical practice, and is presently considered the best marker of ovarian reserve [7], being also related to the live birth rate in IVF [6]. AMH is a dimeric glycoprotein belonging to the transforming growth factor-beta (TGF-β) superfamily and is produced by the granulosa cells of small preantral follicles [8]. Differently from FSH and AFC, AMH is a direct expression of the amount of small follicles that constitute the bulk of ovarian reserve [9,10]. Furthermore, its serum levels show a very low inter- and intra-cycle variability and are independent of the menstrual cycle phase [11].

At present, however, automated assay methods for AMH are not yet available, and the Elisa assays currently in use have a rather high variability [12]. These assays are expensive and not yet available in all IVF labs; their cost limits the possibility of using them in many developing countries, where relevant efforts are made to keep IVF programs as cheap as possible.

When a patient has been submitted at least once to ovarian stimulation for IVF, ovarian sensitivity index (OSI) can be easily calculated dividing the total administered gonadotropin dose by the number of oocytes retrieved at OPU. This ratio represents indeed the ovarian resistance to gonadotropins, as the lower is FSH dose, the higher is ovarian sensitivity. However, the term "ovarian sensitivity index" appears to be more immediate than "ovarian resistance" in recalling the idea of ovarian sensitivity, and therefore has been adopted herein.

The present study shows that OSI displays a strong, inverse correlation with AMH levels, and that this correlation is stronger than those between AMH and the total gonadotropin dose, or between AMH and the total number of retrieved oocytes. This is not surprising as it was shown that neither the total FSH dose administered, nor the absolute number of oocytes accurately reflect ovarian responsiveness to hormonal stimulation; their ratio is much more accurate in describing how the ovary produces oocytes in response to exogenous stimulation [13].

According to our observations, OSI does not accurately reflect the patient's age, even if it shows a fairly good correlation with it (r = 0.40). It rather appears to be partially independent on age: this suggests that it could be incorporated in prediction models aimed at predicting ovarian responsiveness to exogenous gonadotropins as a partially independent parameter.

Our data have been obtained using the GnRH-agonist buserelin plus rFSH in a classical "long" protocol; it must be remarked that the correlation between OSI and AMH found herein could be slightly different in case a different stimulation schedule or different drugs are used. It has been reported, in fact, that rFSH stimulation yields on the average more oocytes with a lower dose compared to what happens using human menopausal gonadotropin (hMG) [14].

In conclusion, the present study shows that OSI accurately reflects AMH level of women submitted to IVF with a "long" protocol. According to the present study, OSI appears to be a highly reliable index of ovarian responsiveness to rFSH and can be useful to estimate the rFSH dose in all IVF cycles following the first, provided that the same kind of protocol is used. We have studied herein a relatively small cohort of patients, although quite homogeneous: obviously larger scale (and maybe multicenter) studies are required to conclude that OSI may be used as a surrogate for AMH for predicting the ovarian response in IVF. The possibility that OSI could be a reliable surrogate of AMH measurement is particularly interesting for IVF Units where AMH assay is not yet available or is considered too expensive to be introduced, e.g. in developing countries where the economical cost of IVF program needs to be limited.

## Competing interests

The authors declare that they have no competing interests.

## Authors' contributions

VB, AP and AR conceived the study, participated in its design and coordination, wrote the manuscript. AB carried out the laboratory assays. PD performed the statistical analysis. CM and CB critically reviewed the study and helped to draft the manuscript. All authors read and approved the final manuscript.
